# Unveiling Allosteric Regulation and Binding Mechanism of BRD9 through Molecular Dynamics Simulations and Markov Modeling

**DOI:** 10.3390/molecules29153496

**Published:** 2024-07-25

**Authors:** Bin Wang, Jian Wang, Wanchun Yang, Lu Zhao, Benzheng Wei, Jianzhong Chen

**Affiliations:** 1 Center for Medical Artificial Intelligence, Shandong University of Traditional Chinese Medicine, Qingdao 266112, China; wb1696843361@gmail.com; 2 School of Science, Shandong Jiaotong University, Jinan 250357, China; wangjian_lxy@sdjtu.edu.cn (J.W.); yangwch1982@126.com (W.Y.); zhaolusdu@163.com (L.Z.)

**Keywords:** BRD9, molecular dynamics simulations, Markov models, binding free energy

## Abstract

Bromodomain-containing protein 9 (BRD9) is a key player in chromatin remodeling and gene expression regulation, and it is closely associated with the development of various diseases, including cancers. Recent studies have indicated that inhibition of BRD9 may have potential value in the treatment of certain cancers. Molecular dynamics (MD) simulations, Markov modeling and principal component analysis were performed to investigate the binding mechanisms of allosteric inhibitor POJ and orthosteric inhibitor 82I to BRD9 and its allosteric regulation. Our results indicate that binding of these two types of inhibitors induces significant structural changes in the protein, particularly in the formation and dissolution of α-helical regions. Markov flux analysis reveals notable changes occurring in the α-helicity near the ZA loop during the inhibitor binding process. Calculations of binding free energies reveal that the cooperation of orthosteric and allosteric inhibitors affects binding ability of inhibitors to BRD9 and modifies the active sites of orthosteric and allosteric positions. This research is expected to provide new insights into the inhibitory mechanism of 82I and POJ on BRD9 and offers a theoretical foundation for development of cancer treatment strategies targeting BRD9.

## 1. Introduction

The complexity and therapeutic resistance of cancers have long posed significant challenges to medical research and human health. Among various factors influencing cancer development, epigenetic changes play a crucial role, particularly acetylation modification of histones [1,2]. This modification not only regulates gene transcription activity but is also closely associated with the onset and progression of various cancers. The bromodomain (BRD) family proteins, acting as epigenetic “readers” capable of recognizing acetylated histones [3,4], have emerged as novel targets for cancer therapy. BRD family proteins are evolutionarily conserved epigenetic reader modules that recognize N-acetylated lysine (KAc) residues on histones and other proteins [5]. These histone tail modifications are involved in controlling chromatin accessibility. The motifs recognized by BRD family proteins work in concert with other chromatin factors to regulate gene transcription [6,7].

To date, the BRD family has been classified into eight subfamilies [8,9,10], including at least 56 nuclear or cytoplasmic proteins, in humans, with diverse structures and functions. Furthermore, there is growing evidence suggesting that BRD family members play a role in the pathogenesis of various diseases by modulating the transcription of multiple genes associated with cancer growth and inflammation [11,12,13,14]. Consequently, BRD family members have been potential targets for anti-cancer drug design, with several BRD inhibitors in development and some even in clinical trials for both oncological and non-oncological conditions.

**Figure 1:**
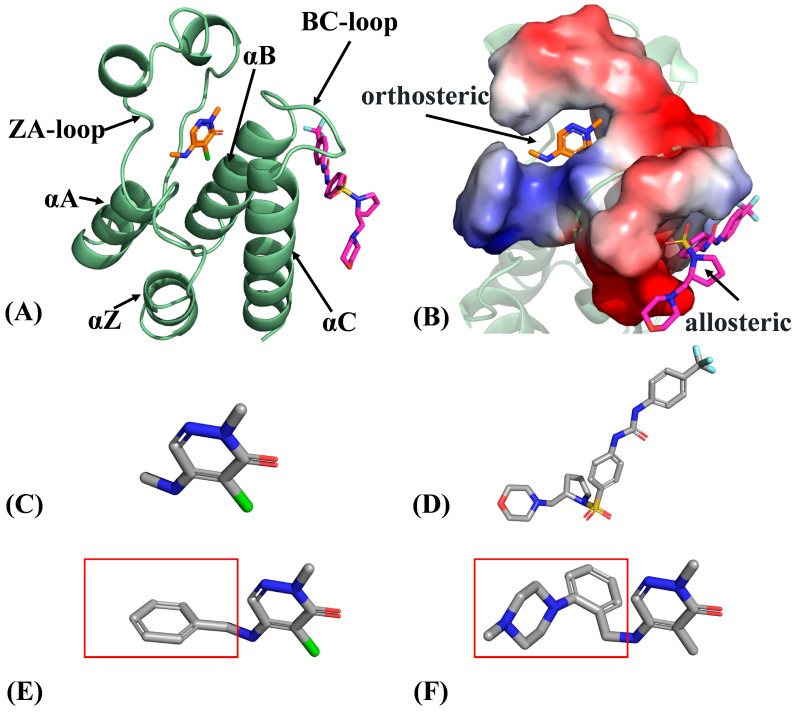
Molecular structures: (**A**) structural superimposition of inhibitor-bound BRD9 complex, (**B**) binding pocket of orthosteric site and allosteric site, (**C**) orthosteric inhibitor 82I, (**D**) allosteric inhibitor POJ, (**E**) orthosteric inhibitor compound 9 (named as LIG) and (**F**) orthosteric inhibitor P8Z. In this figure, the boxes illustrate the differences among molecular groups. The compound 9 was taken from the work of Clegg et al [22].

Despite significant sequence variation among various BRD family members, all BRD proteins share a common topology [15,16,17], including four left-handed antiparallel α-helices (αZ, αA, αB and αC) connected by functional loop regions, the ZA loop and BC loop, which are responsible for substrate specificity (Figure 1A). Binding pockets of BRD family proteins are depicted in Figure 1B. Among the BRD family members, BRD4 is one of the most extensively studied proteins and has been shown to play a crucial role in various cancers [18,19,20]. In recent years, the development of allosteric inhibitors targeting BRD4, such as ZL0590 (also known as POJ), have proven to be interesting in new therapeutic approaches of cancers [21]. ZL0590, as a novel allosteric inhibitor, binds to a new site on BRD4 that is different from the binding pocket of traditional acetylated lysine and possesses a specific binding mode and therapeutic potential. Insights into binding of ZL0590 to BRD proteins are of importance for design of allosteric inhibitors toward BRD family members.

Similar to BRD4, BRD9 also belongs to the BRD family, and its role in tumor development is gaining special attention. Since BRD9 is also involved in the regulation of chromatin remodeling and gene expression, it significantly impacts the proliferation and survival of tumor cells [23,24,25,26]. Currently, multiple investigations have been performed to probe the binding mechanism of inhibitors to BRD9 and the conformational alterations of BRD9 caused by inhibitor binding [22,27,28,29,30]. Recently, an orthosteric inhibitor, 4-chloro-2-methyl-N-methylaminopyridine (also known as 82I), has been identified as an atypical acetyl-lysine methyl mimic and can bind to the active site of BRD9, showing an inhibitory activity with an IC50 of 25.1 μM against BRD9 [22]. Figure 1C shows the molecular structure of 82I. The development of this orthosteric inhibitor provides a new strategy for treatment of BRD9-related diseases. While the allosteric inhibitor ZL0590 has shown high affinity and efficacy on BRD4, as depicted in Figure 1D [31], its impact on BRD9 remains unclear. To obtain more experimental information, two additional orthosteric inhibitors, compound 9 (named as LIG) with an IC50 value of 2000 nM and P8Z with an IC50 value of 200 nM, were used to compare their binding information and probe the effects of structural differences on inhibitor binding [22]. Therefore, it is highly necessary to probe the molecular mechanism of orthosteric and allosteric regulations on BRD9’s activity for the development of anti-cancer drugs targeting BRD9.

To reach our aims, orthosteric inhibitor 82I and allosteric inhibitor POJ were selected for this work to study the orthosteric/allosteric effect, and two orthosteric inhibitors P8Z and LIG were used to test the reliability of our results. Molecular dynamics simulations [32,33,34,35] and free energy analyses [36,37,38,39,40,41] have been extensively adopted to decode the regulation mechanism of small molecules on the target activity. Multiple independent molecular dynamics (MIMD) simulations [42,43], followed by Markov modeling and associated analyses [44], were performed to explore the various macro-states transitions of BRD9 induced by the association of inhibitors. Time-lagged independent component analysis (TICA) [45,46,47] and principal component analysis (PCA) [48,49,50,51] were combined to explore changes in conformational space of BRD9 due to inhibitor binding. Flux analysis was carried out to reveal transition probabilities between different macro-states of BRD9 and clarify the corresponding structural information [52,53,54]. In addition, molecular mechanics-generalized Born surface area (MM-GBSA) and solvated interaction energy (SIE) methods were applied to calculate the binding free energies of 82I, LIG, P8Z and POJ to BRD9 and understand their binding abilities. We also anticipate that this work can contribute useful information to the development of potent anti-cancer drugs for the BRD family.

## 2. Results and Discussion

### 2.1. Structural Stability and Flexibility 

**Figure 2:**
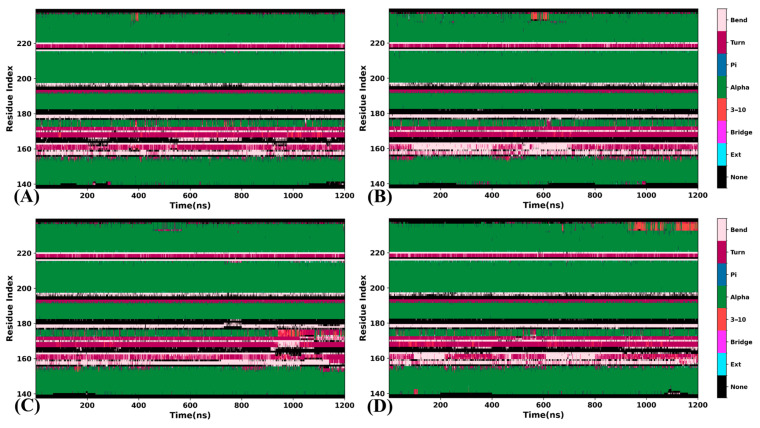
Secondary structure evolution of BRD9 in four systems as the simulation time: (**A**) the APO-BRD9, (**B**) the 82I-BRD9, (**C**) the POJ-BRD9 and (**D**) the ALL-BRD9.

To evaluate the effect of orthosteric and allosteric regulation on structures of BRD9, RMSDs of all the six systems were calculated by using the coordinates of Cα atoms relative to the crystal structure. Their time course and distributions are depicted in supporting information, with Figure S1A–C showing the analyses of four systems, including BRD9 without the inhibitor binding (APO-BRD9), 82I-bound BRD9 (82I-BRD9), POJ-bound BRD9 (POJ-BRD9) and both the 82I and POJ-bound BRD9 (ALL-BRD9), and Figure S2A–C showing the 82I-, LIG-, P8Z and POJ-BRD9 systems. Overall, the systems share similar structural fluctuations (Figures S1 and S2). According to the RMSD distributions (Figure S1B,C), binding of orthosteric and allosteric inhibitors does not produce an obvious influence on the structural stability of BRD9. Differently, binding of LIG and P8Z decreases the RMSD of BRD9 (Figure S2B), indicating that the presence of these two inhibitors is favorable for the stabilization of the BRD9 structure. Additionally, the time evolution of secondary structures from BRD9 in the systems was computed to evaluate the inhibitor-induced effect on secondary structures (Figure 2 and Figure S3). Except for interconversion between turns and bends formed by residues 155 to 165, secondary structures of BRD9 are stable throughout the entire MD simulations, which possibly facilitates the function of BRD9. It is worth noting that binding of only the allosteric inhibitor leads to the evolution of the helix into the 3–10α after ~950 ns while in ALL-BRD9 the helix is well maintained, indicating that binding of an allosteric inhibitor can lead to a different effect compared orthosteric inhibitors.

The radius of gyrations (Rgs) for the APO-, 82I-, POJ- and ALL-BRD9 were determined to reveal changes in structural compactness of BRD9 during the simulations, and their time courses and distributions are displayed in Figure S1D–F. Meanwhile, the radius of gyrations (Rgs) for the 82I-, LIG-, P8Z- and POJ-BRD9 are depicted in Figure S2D–F. It is noted that the Rgs of BRD9 in the systems possess similar fluctuation range (Figures S1D and S2D); moreover, the presence of orthosteric and allosteric inhibitors hardly impacts the structural compactness extents of BRD9 (Figures S1E,F and S2E,F). At the same time, the molecular surface area (MSA) of BRD9 from six systems was computed to probe the effect of orthosteric and allosteric regulation on the solvent-accessible extents of BRD9. Figures S1G and S2G show the evolution of MSA over the simulation time, and Figures S1H,I and S2H,I display the MSA distribution. It is observed that the MSAs of 82I-BRD9, POJ-BRD9, ALL-BRD9, LIG-BRD9 and P8Z-BRD9 were reduced by 150, 250, 295, 150 and 250 Å2 relative to APO-BRD9, respectively, indicating that the binding of orthosteric and allosteric inhibitors decreases the contacting extents of the solvent with BRD9, in particular for ALL-BRD9. This result implies that the presence of inhibitors in orthosteric and allosteric positions of BRD9 affects the activity of BRD9.

**Figure 3:**
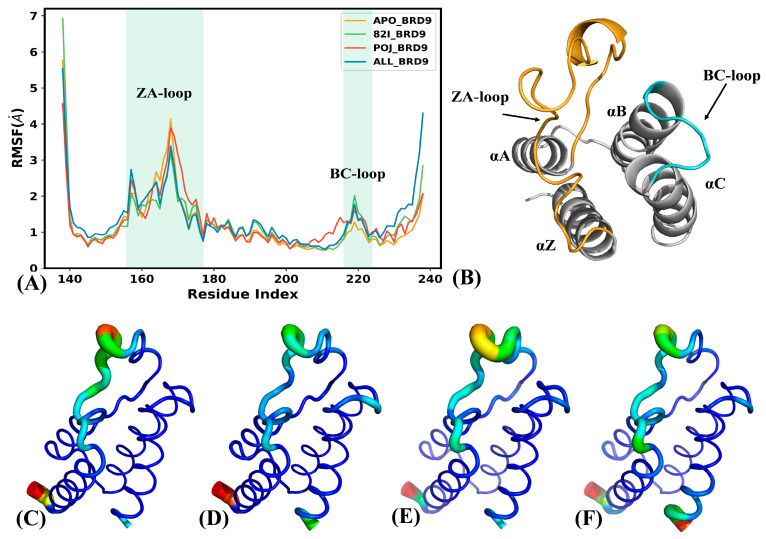
Structural flexibility of the APO-, 82I-, POJ-, ALL-BRD9: (**A**) the RMSFs of BRD9 calculated using the coordinates of the Cα atoms, (**B**) structural regions with obvious alterations of RMSFs and (**C**–**F**) corresponding to the structural flexibility of APO-BRD9, 82I-BRD9, POJ-BRD9 and 82I/POJ-BRD9. The tendency from blue to red indicates the increase in structural flexibility which is scaled in B-factor.

To understand the influence of orthosteric and allosteric regulations on the structural flexibility of BRD9, RMSFs of BRD9 were computed using the coordinates of the Cα atoms (Figure 3A and Figure S4A), and the structural regions showing obvious alterations in RMSFs are exhibited in Figure 3B and Figure S4B. As observed in Figure 3A, the ZA-loop of BRD9 shows high flexibility while the BC-loop has a weaker flexibility compared to the ZA-loop (Figure 3B). By comparison with APO-BRD9, the association of orthosteric and allosteric inhibitors weakens the structural flexibility of the ZA-loop, particularly in the case of ALL-BRD9 (Figure 3A). On the contrary, binding of 82I and POJ slightly strengthens the structural flexibility of the BC-loop relative to the APO-BRD9, especially when both inhibitors are bound (ALL-BRD9) (Figure 3A,B). Compared to the POJ-BRD9, binding of three orthosteric inhibitors (82I, LIG and P8Z) leads to a more rigid ZA-loop (Figure S4A). In addition, the B-factor of the Cα atoms was also estimated and visualized using the PyMOL program to clarify the inhibitor-mediated changes in flexibility for BRD9. The results are depicted in Figure 3C–F. It is observed that the flexibility of the ZA-loop was decreased by inhibitor binding compared to the APO-BRD9, which is in good agreement with the RMSF analysis. At the same time, the structural flexibility of the ZA-loop in BRD9, when bound by 82I, LIG and P8Z, is reduced relative to POJ-BRD9 (Figure S4C–F).

In summary, binding of orthosteric and allosteric inhibitors exerts certain impacts on the solvent-accessible extents and structural flexibility of BRD9. The structural flexibility of the ZA-loop was suppressed due to the binding of orthosteric and allosteric inhibitors compared to the APO-BRD9, while that of the BC-loop was enhanced by the associations of two types of inhibitors. Moreover, the presence of double inhibitors (ALL-BRD9) produces a more obvious effect on the flexibility of these two loops than the binding of a single inhibitor. These changes imply that orthosteric and allosteric inhibitors can regulate the activity of BRD9, and our current findings agree well with the previous work [22,28].

### 2.2. Internal Dynamics of BRD9 Affected by Binding of 82I and POJ

**Figure 4:**
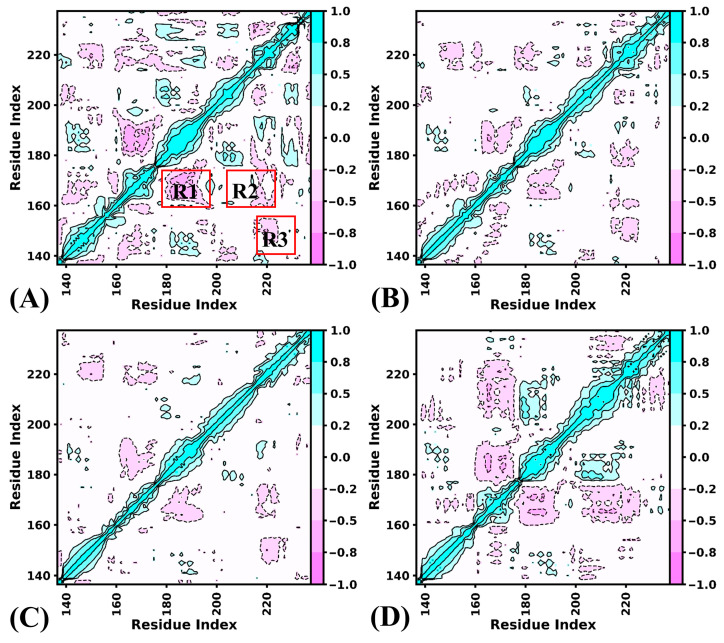
DCCMs of BRD9 calculated by using the coordinates of the Cα atoms: (**A**) the APO-BRD9, (**B**) the 82I-BRD9, (**C**) the POJ-BRD9 and (**D**) the ALL-BRD9. The red-colored R1, R2, and R3 regions represent the primary differences among the four systems.

To clarify inhibitor-mediated impacts on internal dynamics of BRD9, the DCCMs were computed using the coordinates of the Cα atoms with the CPPTRAJ program, and the results are presented in Figure 4, in which cyan and purple indicate strong positive correlations in motion and anti-correlation movements, respectively. It is noted that binding of orthosteric and allosteric inhibitors mainly affect correlation of movements of three regions: including the R1 (parts of the ZA-loop: residues 178–193), R2 (the parts of the BC-loop and the C-terminal of αB: residues 204–221) and R3 (the parts of the BC-loop and the N-terminal of αC). For the APO-BRD9, the regions R1 and R2 yield anti-correlated motions relative to residues 159–174, while the region R3 generates weak anti-correlation movement relative to residues 123–154 (Figure 4A). By comparison with the APO-BRD9, inhibitor binding enhances the anti-correlation movements of R1 and R2 relative to residues 159–174, particularly in the binding of the allosteric inhibitor POJ (Figure 4B–D). Binding of the orthosteric inhibitor 82I increases the anti-correlation motion of R3 compared to APO-BRD9, while the cooperation of 82I and POJ leads to stronger anti-correlation motion in R3 relative to APO-BRD9 and 82I-BRD9 (Figure 4B,D). By comparison with BRD9 bounded by three orthosteric inhibitors 82I, LIG and P8Z, binding of POJ strengthens the anti-correlation motions of R2 and results in the disappearance of anti-correlation motions in the R3 region (Figure S5). Binding of POJ obviously enhances the anti-correlated motion of the R1 region relative to the LIG- and P8Z-BRD9 but hardly changes the correlated movements of this region compared to 82I-BRD9 (Figure S5). According to structural information, R1 and R2 are involved in the orthosteric regulation of BRD9, while region R3 is involved in the allosteric regulation of BRD9.

**Figure 5:**
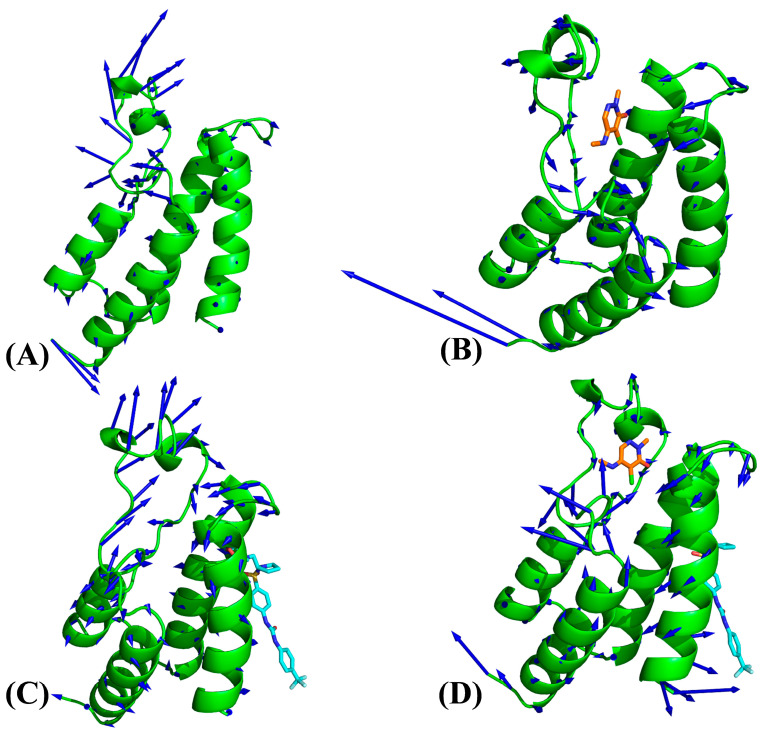
Concerted motions of structural domains in four systems: (**A**) the APO-BRD9, (**B**) the 82I-BRD9, (**C**) the POJ-BRD9 and (**D**) the ALL-BRD9. In this figure, BRD9 is shown in cartoon modes and inhibitors are displayed in stick modes.

To reveal the influence of orthosteric and allosteric inhibitors on the concerted motions of BRD9, the first eigenvector from PCA was visualized using the PyMOL program with the initialized structure (Figure 5 and Figure S6). The ZA-loop of APO-BRD9 shows more disordered motions, represented by the length of the arrows (Figure 5A), and meanwhile the BC-loop has an outward motion tendency (Figure 5A). Compared to APO-BRD9, binding of a single orthosteric 82I not only inhibits the structural fluctuation of the ZA-loop along the first eigenvector but also induces an inward movement tendency of the BC-loop (Figure 5B). By referencing APO-BRD9, binding of a single allosteric inhibitor POJ leads to highly concerted fluctuations along the first eigenvector and mediates an inward motion tendency of the BC-loop (Figure 5C). Through comparison with APO-BRD9, the cooperation of orthosteric and allosteric inhibitors not only greatly inhibits the fluctuations of the ZA-loop along the first eigenvector but also induces a downward motion tendency of the BC-loop (Figure 5D). By referencing BRD9 complexed with three orthosteric inhibitors, the binding of POJ and the disappearance of 82I, LIG and P8Z from the orthosteric pocket not only strengthens the fluctuations of the ZA-loop and BC-loop along the PC1 direction but also changes fluctuation tendencies of these two loops (Figure S6).

By combining the above analyses, the binding of orthosteric and allosteric inhibitors obviously affects the correlated motions and concerted movements of the ZA-loop and BC-loop. The ZA-loop and BC-loop are involved in the binding pocket at the orthosteric position, while the BC-loop also takes part in the formation of the allosteric binding pocket. Thus, the changes in correlated motions and concerted movements of these two loops caused by 82I and POJ are likely to modulate the function of BRD9 and its activity. The previous works also indicated that inhibitor binding produced a significant effect on the function of BRD9 [13,22], which supports our current findings.

### 2.3. Analyses of Markov Model

**Figure 6:**
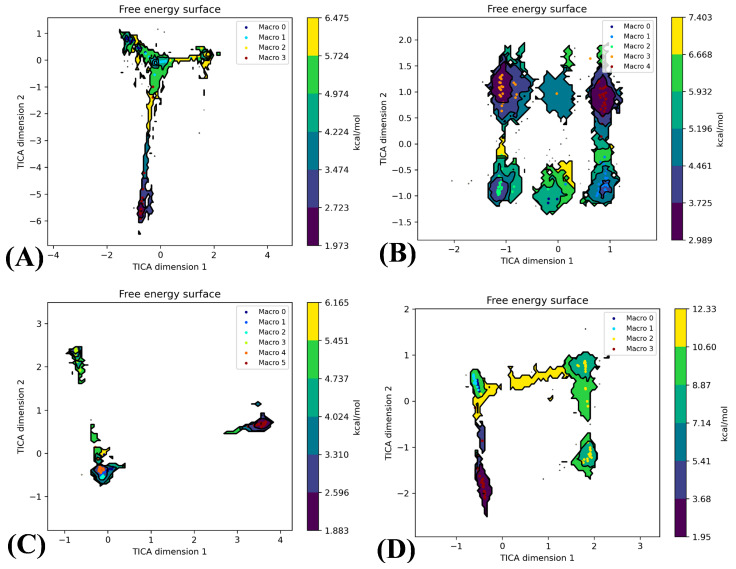
Free energy surface of four systems arising from the TICA: (**A**) the results of k-means diagrams of the APO-BRD9, (**B**) the results of k-means diagrams of the 82I-BRD9, (**C**) the results of k-means diagrams of the POJ-BRD9 and (**D**) the results of k-means diagrams of the ALL-BRD9.

To assess macrostates and microstates of inhibitor-bound BRD9, a Markov model was employed to analyze MD trajectories, incorporating techniques such as TICA, the k-means clustering algorithm, lag time calculations and flux analysis using the HTMD package [55]. For the under-study systems, APO-, 82I-, POJ- and ALL-BRD9, three independent molecular trajectories were transformed into three two-dimensional arrays with dimensions of 600,000 × 101. The first dimension of the two-dimensional array (600,000) corresponded to the conformation numbers of the three independent trajectories, while the second dimension (101) represented the residue numbers. Then, TICA was performed using the k-means algorithm to capture low-frequency motions, with a k-value of 100 selected through the clustering procedure, as depicted in Figure 6. The analysis revealed that APO-BRD9, 82I-BRD9, POJ-BRD9 and ALL-BRD9 have 4, 5, 6 and 4 macrostates, respectively. A main macrostate of APO-BRD9, two main macrostates of 82I-BRD9, a main macrostate of POJ-BRD9 and a main macrostate of ALL-BRD9 account for 77.4%, 81.4%, 67.6% and 98.1% of the total sampling times, respectively (Figure S7). These results imply that the cooperation of orthosteric and allosteric inhibitors stabilizes the conformations of BRD9.

**Figure 7:**
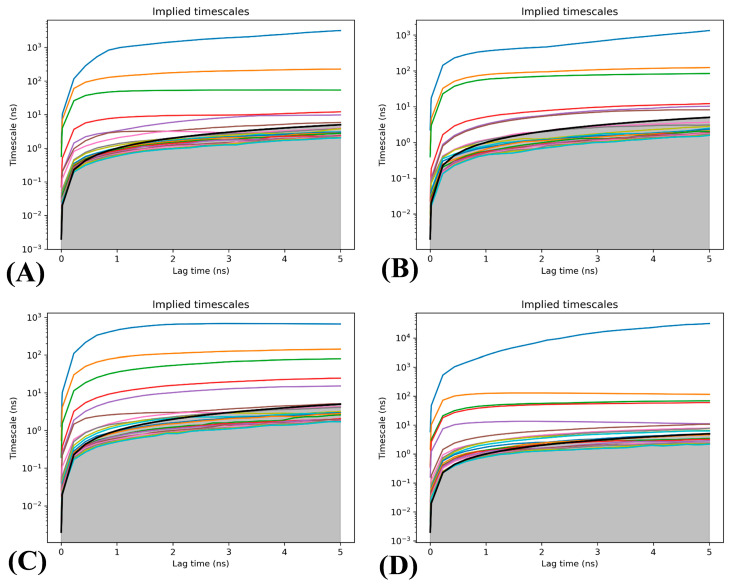
Lag time determination of four systems: (**A**) the APO-BRD9, (**B**) the 82I-BRD9, (**C**) the POJ-BRD9 and (**D**) the ALL-BRD9. In the figure, each curve’s color indicates a different implied timescale, and the black line with the gray shading below shows the model’s resolution limit.

To more efficiently construct Markov models, the lag times of the systems were computed through the aforementioned clustering results arising from the TICA, and the results are displayed in Figure 7. The minimum lag time, which is the Markov time, is identified when the relaxation time scale reaches convergence. For the APO-BRD9 and POJ-BRD9, the relaxation time scales tend to converge when the lag time is 1 ns (Figure 7A,C). With respect to the 82I-BRD9 and ALL-BRD9, the relaxation time scales tend to converge when the lag time is 4 ns (Figure 7B,D). Compared to the APO-BRD9 and POJ-BRD9, the presence of the orthosteric inhibitor 82I results in a longer convergence lag time, indicating that orthosteric inhibitors can better stabilize the BRD9 conformation relative to allosteric inhibitors.

**Figure 8:**
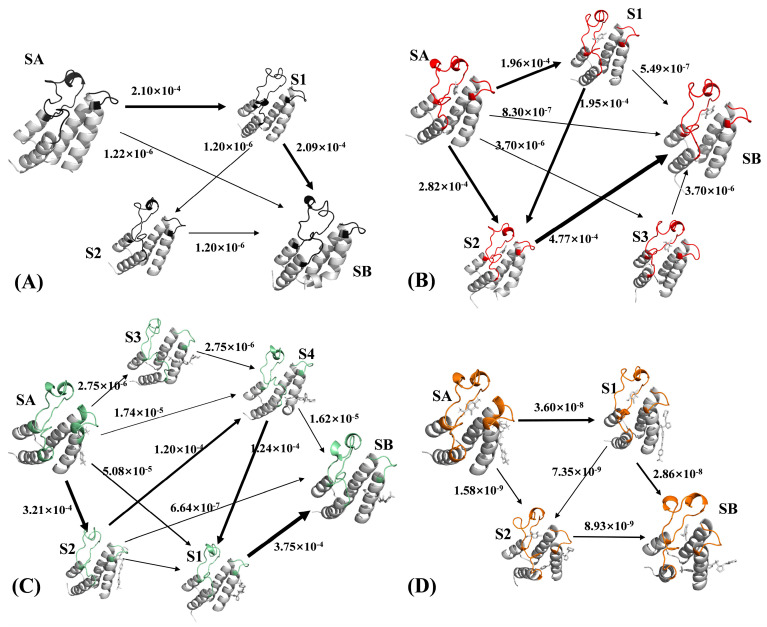
Flux analysis of four simulation systems: (**A**) the APO-BRD9, (**B**) the 82I-BRD9, (**C**) the POJ-BRD9 and (**D**) the ALL-BRD9.

**Figure 9:**
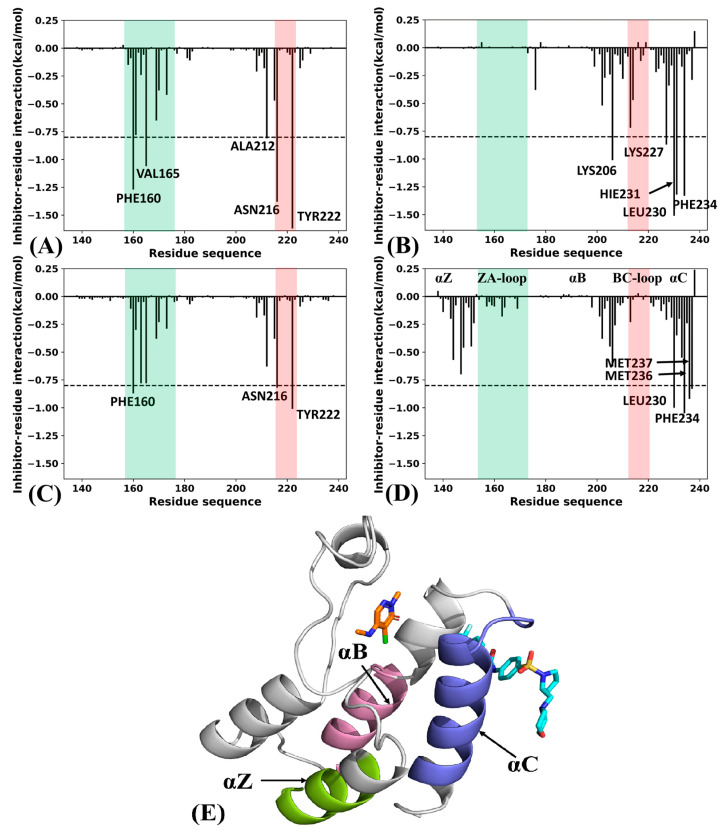
Inhibitor–residue interactions: (**A**) the 82I-residue interactions in the 82I-BRD9, (**B**) the POJ-residue interactions in the POJ-BRD9, (**C**) the 82I-residue interactions in the ALL-BRD9, (**D**) the POJ-residue interactions in the ALL-BRD9 and (**E**) the orthosteric and allosteric binding pockets.

Based on obtained various macroscopic states, flux analysis was carried out to compute the transition probability matrix of the Markov model, which is visually shown in Figure 8 with its proportion of each path. Meanwhile, the same information regarding the flux analysis was also listed at Table 1 and Tables S1–S3. For the 82I-BRD9, the SA → S1 → SB is a main conformational transition path (Figure 8A), and this transition accounts for 98.9% of the total flux, while the other transitions only account for 1.1% of the total flux. Compared to the APO-BRD9, binding of orthosteric inhibitor 82I induces two primary transition pathways SA → S2 → SB and SA → S1 → S2 → SB (Figure 8B), respectively accounting for 58.4 and 40.5% of the total flux (Table S1). This result implies that the presence of orthosteric inhibitors leads to conformational rearrangement of BRD9. By referencing the APO-BRD9, the binding process of allosteric inhibitor POJ mediates four main transition pathways, including SA → S2 → S1 → SB, SA → S2 → S4 → S1 → SB, SA → S1 → SB and SA → S4 → SB (Figure 9C). They account for 51.1, 30.6, 13.0 and 4.1% of the total flux (Table S2), individually, showing that binding of the allosteric inhibitor induces more energy states than the APO-BRD9 and the activity of BRD9 can be regulated through the allosteric inhibitor. By comparison with the APO-BRD9, three key transition pathways are detected in the ALL-BRD9, consisting of SA → S1 → SB, SA → SB and SA → S1 → S2 → SB (Figure 8D), while they separately account for 76.2, 19.6 and 4.2% of the total flux. This result suggests that the cooperation of orthosteric and allosteric inhibitors can regulate the activity of BRD9 by altering the conformational transition pathway.

Based on the current analyses of the Markov model, binding of a single orthosteric inhibitor, allosteric inhibitor, or the cooperation of two types of inhibitors can exert different effects on the distributions of macrostates and microstates as well as the conformational transition pathway, which regulate the activity of BRD9. The distances between the ZA-loop and BC-loop indicate that 82I, POJ and the cooperation of two types of inhibitors yield different effects on the conformations of these two loops (Figure S8), basically supporting the results of our current Markov model analysis. In addition, the work on BRD4 from Yang et al. verified that the binding of orthosteric and allosteric inhibitors can tune the activity of BRD4 [13], which partially supports our work.

### 2.4. Binding Ability of Two Types of Inhibitors to BRD9

Binding free energies are regarded as an important indicator for measuring binding ability of inhibitors to targets. Because the experimental information on the binding of POJ in the allosteric sites of BRD9 is lacking, we used three different methods, including molecular docking, MM-GBSA and SIE methods, to calculate binding free energies so as to evaluate the reliability of our calculations. MM-GBSA calculations were performed on 300 snapshots selected from the equilibrated parts of the MD trajectory under consideration. To reduce high computation time, 100 snapshots from the aforementioned 300 were applied to estimate the entropy contributions. The same snapshots used in the MM-GBSA calculations were utilized to perform the SIE calculations.

The inhibitors 82I, LIG and P8Z were docked into the orthosteric pocket of BRD9 while POJ was docked into the allosteric pocket and the binding data was provided in Table S4, with the structures of the first ten docking scores highlighted in blue (Figure S9). According to Table S4, binding ability of four inhibitors scaled by the best scoring are in the order POJ < 82I < LIG < P8Z. The binding poses of the four inhibitors indicate that 82I, LIG and P8Z with higher scores mostly bind at the orthosteric position while POJ, with higher scores, is mainly found in the allosteric pocket of BRD9 (Figure S9). These results confirm the reliability of the docked structures of 82I, LIG, P8Z and POJ used for MD simulations.

Table S5 lists the results calculated by the MM-GBSA method, and Table S6 provides the data estimated by the SIE method. Firstly, the ranks of binding free energies of 82I, LIG and P8Z predicted by two methods are in good agreement with those determined by the experimental values. Secondly, the ranks of binding ability for 82I, LIG, P8Z and POJ to BRD9 predicted by the previous molecular docking are in good consistency with those of the four inhibitors to BRD9 calculated by MM-GBSA and SIE methods. These results further verify that our current free energy analyses are reliable and valid.

Binding free energies of 82I and POJ in the three bound states, single 82I, single POJ and the ALL state, are shown in Table 2. The electrostatic interactions ($\Delta E_{ele}$), van der Waals interactions ($\Delta E_{vdW}$) and non-polar solvation free energies ($\Delta G_{surf}$) contribute favorable forces to associations of 82I and POJ with BRD9 while polar solvation free energies ($\Delta G_{gb}$) and the entropy effect (−T∆S) are unfavorable for the binding of these two inhibitors (Table 2). Binding free energies of 82I to BRD9 in the 82I-BRD9 and ALL-BRD9 states are −4.83 and −1.31 kcal/mol, respectively, and that of POJ to BRD9 in the POJ-BRD9 and ALL-BRD9 states are −2.42 and −3.59 kcal/mol, individually (Table 2). The binding ability of 82I to BRD9 in the ALL-BRD9 is weakened by 3.52 kcal/mol relative to the 82I-BRD9, but that of POJ to BRD9 in the ALL-BRD9 is strengthened by 1.17 kcal/mol compared to the POJ-BRD9. This result implies that the cooperation of orthosteric and allosteric inhibitors can affect their binding ability to BRD9 and efficiently regulate the activity of BRD9, which is useful for the design of novel inhibitors targeting BRD9 and even the BRD family.

To determine the roles of separate residues in the binding of the two types of inhibitors, inhibitor–residue interactions were calculated through the residue-based free energy decomposition method (Figure 9 and Figure S10). According to Figure 9A and Figure S10A, the active sites of orthosteric inhibitor 82I mainly involve residues PHE160, VAL165, ALA212, ASN216 and TYR222 in the 82I-BRD9, and their interaction energies with 82I are more negative than −0.8 kcal/mol. As shown in Figure S10B,C, the two orthosteric inhibitors LIG and P8Z share binding clusters of residues that are the same as those in 82I, implying orthosteric inhibitors possess a similar binding cavity. By comparison with the three orthosteric inhibitors, allosteric inhibitor POJ loses the interaction with the first residue cluster labeled in green (Figure S10D). Compared to the 82I-BRD9, the cooperation of two types of inhibitors leads to decreases in the interaction energies of PHE160, VAL165, ALA212, ASN216 and TYR222 with 82I in the ALL-BRD9 (Figure 9C). As shown in Figure 9B and Figure S10D, the active sites of allosteric inhibitor POJ primarily include residues LYS206, LYS227, LEU230, HIE231 and PHE234 in the POJ. Moreover, the interaction energies of these five residues are more negative than −0.8 kcal/mol. Although the cooperation of the two types of inhibitors weakens the interactions of LYS206, LYS227, LEU230, HIE231 and PHE234 with POJ compared to the POJ-BRD9 (Figure 9D), it strengthens the interactions of MET236 and MET237 with POJ. Meanwhile, the cooperation of the two types of inhibitors also induces additional interaction clusters of residues (the helix αZ) with POJ relative to the POJ-BRD9 (Figure 9B,D), which strengthens the binding ability of POJ to BRD9 in the ALL-BRD9. Thus, the cooperation of the two types of inhibitors in the orthosteric and allosteric positions (Figure 9E) can efficiently regulate the activity of BRD9. The orthosteric active sites revealed by our current work, including PHE160, PHE163, VAL165, ILE169, ASN216 and TYR222, agree well with the information uncovered by previous works [22], implying the reliability of our results.

Based on the above analyses, the cooperation of the two types of inhibitors produces a significant effect on the activity of BRD9: (1) the cooperation of 82I and POJ weakens the binding ability of orthosteric inhibitor 82I to BRD9 and strengthens that of allosteric inhibitor POJ to BRD9, (2) the cooperation of 82I and POJ changes the active sites at the orthosteric and allosteric positions, and (3) van der Waals interactions are the main forces of the two types of inhibitors to BRD9. Thus, more attention should be paid to van der Waals interactions in the future design of orthosteric and allosteric inhibitors.

## 3. Materials and Methods

In this study, we employed a suite of computational biology methods to investigate the dynamics and functions of proteins. Our methodology started with molecular docking, integrated with three-dimensional structural information obtained from the Protein Data Bank (PDB) to construct a series of distinct system models. Specifically, we built apo systems, orthosteric systems (including 82I, P8Z and LIG systems), allosteric systems (POJ system) and dual-inhibitor systems (ALL system). These systems were subsequently subjected to refined processing through molecular dynamics simulations (MD) to acquire in-depth dynamic structural information.

Based on the trajectories derived from the simulations, we carried out two main analytical tasks in parallel. First, we conducted an in-depth analysis of the synergistic effects of the orthosteric, allosteric, and dual-inhibitor systems, employing methods such as Markov modeling, molecular mechanics with Poisson–Boltzmann surface area (MM GBSA), and principal component analysis (PCA). Second, to validate the behavioral differences among various systems, we performed comparative verification analysis on multiple orthosteric and allosteric systems, primarily utilizing MM GBSA, Solvated Interaction Energy method (SIE), and molecular docking techniques.

**Figure 10:**
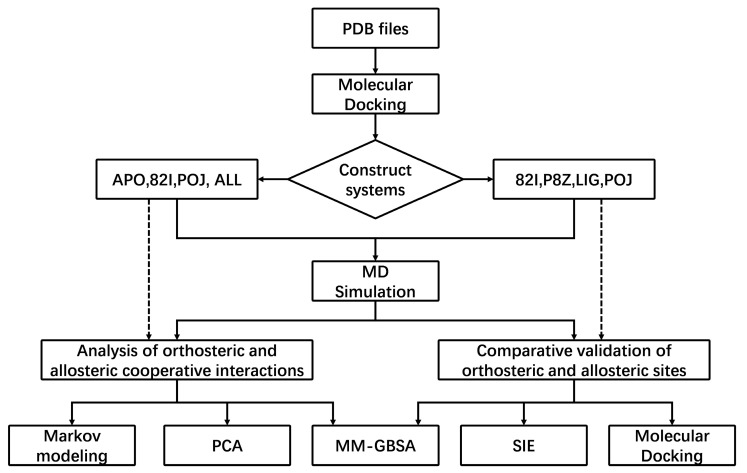
The flowchart of this study, which is used to describe the flow that we performed this study.

The flowchart of this study (see Figure 10) encapsulates the overall framework of the aforementioned methods, clearly depicting the complete pathway from system construction to simulation and then to analysis. This process reflects the logicality of our research design, facilitating the understanding of the computational methods and analytical strategies we employed.

### 3.1. Preparation of Simulation Systems

To investigate molecular mechanism of orthosteric and allosteric regulation, we designed six distinct systems, including the APO form of BRD9 (APO-BRD9) without inhibitor binding, the orthosteric inhibitor 82I/BRD9 complex (82I-BRD9), the allosteric inhibitor POJ/BRD9 complex (POJ-BRD9), and the dual inhibitor/BRD9 complex (ALL-BRD9). In addition, two orthosteric inhibitors, LIG and P8Z, were used for this study to perform information comparison and check the reliability of our results. The initial atom coordinates of 82I-BRD9 and P8Z-BRD9 were obtained from the Protein Data Bank (PDB), and their PDB entries correspond to 6YQW and 6YQS [22]. Since the structures of the POJ-BRD9 and LIG-BRD9 complexes are unavailable in the PDB, the inhibitor POJ [31] and LIG [22] were, respectively, docked into the allosteric binding pocket and orthosteric binding pocket of BRD9 using the HTMD program [55] to generate the structures of the LIG-BRD9 and POJ-BRD9 complexes. The structure of the 82I/POJ-BRD9 (ALL-BRD9) was obtained by removing one BRD9 from the superimposed structures of the 82I-BRD9 and POJ-BRD9. The APO-BRD9 without inhibitor binding was obtained by removing 82I from 6YQW. The H++ 3.0 program [56] was employed to assess protonation states of BRD9 residues and assign reasonable protonation states to each residue from BRD9. The missing hydrogen atoms from the crystal structure were added to the corresponding heavy atom using the Leap module [57,58] in Amber 22. Parameters for BRD9 were derived from the ff19SB force field [59]. The structures of the four inhibitors (82I, LIG, P8Z and POJ) were optimized at the semi-empirical AM1 level, and subsequently, BCC charges were assigned to each atom of the inhibitors using the Antechamber module in Amber [60,61]. The force field parameters for 82I, LIG, P8Z and POJ were obtained using the general Amber force field (GAFF2) [62,63]. Each of the six current systems was solvated in an octahedral periodic box of TIP3P water molecules with a 10.0 Å buffer to mimic the solvent environment, and the force field parameters for water molecules were taken from the TIP3P model [64]. An appropriate number of sodium ions (Na^+^) and chloride ions (Cl^−^) were added to the water box at a 0.15 M concentration of NaCl salt to form neutral simulation systems, with the parameters for Na^+^ and Cl^−^ ions sourced from the research by Joung et al. [65,66].

### 3.2. Multiple Independent Molecular Dynamics Simulations

Initialization of the six BRD9-related systems may lead to high-energy contacts and orientations between atoms throughout the systems, potentially disrupting the stability of the system simulations. To address this issue, all six BRD9-related systems firstly underwent a 5000-cycle steepest descent minimization followed by a 10,000-cycle conjugate gradient minimization. Secondly, in the canonical ensemble (NVT) conditions, the temperature of the six optimized systems was gradually increased from 0 to 300 K over 2 ns, during which all non-hydrogen atoms of the BRD9-related systems were restrained with a weak harmonic restraint of 2 kcal·mol^−1^·Å2. Thirdly, a 3-ns equilibration process was performed at 300 K under the isothermal-isobaric ensemble (NPT) to further optimize systems. A 15-ns NPT simulation was then carried out to maintain the system density at 1.01 g/cm^3^. Finally, three independent 400-ns MD simulations were conducted for six current systems at NVT with periodic boundary conditions (PBC) and the particle mesh Ewald method (PME) [67]. In each independent MD simulation, initial atomic velocities were randomly assigned according to the Maxwell distribution. To facilitate post-processing analysis, the three independent MD trajectories were merged into a single MD trajectory (SMT). Throughout all MD simulations, chemical bonds connecting hydrogen atoms to heavy ones were constrained using the SHAKE algorithm [68]. Langevin dynamics [69] were employed to tune the temperature of the six BRD9-related systems, with which a collision frequency of 2.0 ps^−1^ was adopted. The PME method, in conjunction with an appropriate 12-Å cutoff, was utilized for calculating electrostatic interactions (EIs), and this cutoff was also applied for handling van der Waals interactions (VDWIs). The pmemd.cuda program embedded in Amber 22 was used to run MD simulations and relax the systems [70,71]. Analyses of MD trajectories, including root-mean-square deviations (RMSDs), root-mean-square fluctuations (RMSFs), PCA and dynamics cross-correlation maps (DCCMs) [49], were performed through the CPPTRAJ module in Amber [72]. The VMD [73] and PyMOL (www.pymol.org) programs were employed to show structures and depict figures.

### 3.3. Markov Models

Markov models facilitate the categorization of highly similar conformations recorded in MD trajectories into different microstates. Each microstate matches a state belonging to the Markov model state space. The transition probabilities are used to characterize the transformations between these states, as represented by a transition probability matrix. The Markov model can be used to analyze the dynamic relationships between various microstates by means of the transition probability matrix and flux analysis methods [44]. Our current Markov model mainly involves the following method: TICA [45,46,47], k-means clustering algorithm, lag time calculations and flux analysis, and the details for these methods are clarified as follows.

#### 3.3.1. TICA Dimensionality Reduction Method

TICA is a commonly utilized dimensionality reduction technique in the construction of Markov models. Markov models can efficiently extract information from multiple repeated trajectories through input MD trajectories. High-dimensional data taken from MD trajectories are difficult to use directly. To improve the efficiency of data processing and analyses, the TICA dimensionality reduction method is adopted to treat data and reveal conformational changes of targets. Although TICA cannot analyze the principal component of data, it can detect the coordinates of the maximum autocorrelation at a given lag time. Thus, TICA can effectively extract slow order parameters from molecular dynamics data, which enables it to be an excellent choice for processing molecular simulation data before k-means clustering [74]. In the context of Markov models, TICA identifies the characteristic function and approximate eigenvalues of the underlying Markov operator from the input data, allowing for the estimation of TICA transformation values. These values can then be used to obtain eigenvalues, eigenvectors or project input data onto the slowest TICA components.

#### 3.3.2. K-Means Clustering Algorithm

The k-means clustering algorithm is a prevalent method in machine learning and falls under the category of unsupervised learning techniques. The algorithm’s core objective is to partition an unlabeled dataset, without prior knowledge, into k segments by optimizing an evaluation function. As a non-convex algorithm, k-means is susceptible to local optima. To mitigate this, random initialization is performed multiple times, with the expectation that the optimal solution will be global. In this study, the k-means clustering process was repeated ten times for each k-value to evaluate the results. Determining the hyperparameter k is challenging and can be guided by the elbow method. However, the elbow method is not always effective. Therefore, this study simplifies the process by applying the rationale of the elbow method, conducting a grid search for more reasonable k-values. In Markov models, k-means clustering is primarily used to cluster conformational states in molecular simulation trajectories, with each cluster defined as a microstate in the Markov model, resulting in a discrete trajectory of microstates [75,76]. Subsequently, the Markov model calculates the transition probabilities between microstates based on this discrete trajectory, yielding the transition probability matrix.

#### 3.3.3. Determination of Lag Time

Lag time is a critical parameter in Markov models for determining the transition probability matrix through multiple separate trajectories. The physical interpretation of lag time is the duration of each jump between discrete trajectories. In practice, after obtaining the discrete trajectories of microstates, a lag time jump is performed along these trajectories, recording the jump information in a counting matrix, which is then transformed into a transition probability matrix. Markov time refers to the minimum lag time that ensures Markov, or memoryless, behavior and is achieved by evaluating the system’s loss of dependence on the implied relaxation timescale [52,77]. Lag time significantly impacts Markov models: a too short lag time can lead to substantial differences between eigenvector errors and spectral errors, while an excessively long lag time may introduce significant numerical errors. The Chapman–Kolmogorov test is commonly used to assess the appropriateness of the chosen lag time, comparing the left and right sides of the Chapman–Kolmogorov equation [78]:

(1) $$P(k\tau )=P^{k}(\tau )$$

where P(τ) is the transition probability matrix determined by the lag time *τ*, and *kτ* represents multiples of *τ*. The lag time *τ* is amplified multiple times, and a Markov model is constructed to obtain the transition probability matrix. If the left and right sides of the equation are equal or very close, it indicates that the chosen lag time τ enables the Markov model to exhibit memoryless behavior, which is typically considered appropriate if the difference between the two sides is less than 5% [79].

#### 3.3.4. Flux Analysis

If molecular simulation trajectories have been clustered into macroscopic stable states, flux analysis can be performed. In practice, once the transition probability matrix of the Markov model is obtained, this result can be utilized to acquire thermodynamic and kinetic information of the trajectory. Due to the high complexity of this model, a more coarse-grained model may be employed to provide the same quantitative information more concisely, making it more suitable for flux analysis [53]. The objective of flux analysis is to determine the transition pathways within the microstate space constructed by the Markov model, which requires a probability distribution matrix and the calculation of flux. The central concept involves calculations of the forward probability $q_{i}^{+}$ and the backward probability $q_{i}^{-}$ for each microstate when it is at equilibrium. The calculation methods for $q_{i}^{+}$ and $q_{i}^{-}$ are as follows [54]:

(2) $$q_{i}^{+}=\sum_{j\in B}T_{ij}+\sum_{j\in \bar{A\cup B}}T_{ij}q_{j}^{+}$$

(3) $$q_{i}^{-}=1-q_{i}^{+}$$

in which $T_{ij}$ represents the transition probability between two specified microstates, provided by the transition probability matrix of the Markov model, with *A* and *B* being the two endpoint microstates [52]. After calculating $q_{i}^{+}$ and $q_{i}^{-}$, the effective flux $f_{ij}$ from microstate *i* to microstate j can be determined based on this. The calculation formula is as follows, where $\rho_{i}$ is the Boltzmann probability of state *i*:

(4) $$f_{ij}=\rho_{i}q_{i}^{-}T_{ij}q_{j}^{+}$$

Thus, the net flux from microstate *i* to microstate *j* can be estimated by using the two effective fluxes, $f_{ij}$ and $f_{ji}$:

(5) $$f_{ij}^{+}=max(f_{ij}-f_{ji})$$

Using this method, the net flux between any two microstates can be calculated. For multiple stable states in molecular dynamics simulation, it is only necessary to identify the initial stable state *A* and the final stable state *B* of the trajectory and then determine the intermediate states between the two endpoints *AB* based on the transition probability matrix and the flux between the stable states. Due to the relationships between transition probabilities, intermediate states are not unique and may present multiple scenarios. Each state scenario will determine an occurrence probability based on the flux along its path. The calculation method for path probability is as follows:

(6) $$P_{i}^{A\to B}=\frac{f_{i}^{A\to B}}{\sum_{j}f_{j}^{A\to B}}$$

After calculating the probabilities of each path, the conformational changes of each path and the probability of its occurrence can be used for path analysis. If one wishes to integrate the cluster size of each stable state, which is also known as the frequency of stable state occurrence, with the flux probability, an allocation coefficient can be assigned to each stable state based on the number of times that this state is observed in the path, and the total probability can be calculated by weighting it according to the probability of the path. Ultimately, the path order with the highest probability can be obtained.

### 3.4. MM-GBSA Calculations

MM-PB/GBSA [80] methods have been an efficient tool for calculating inhibitor–target binding free energies. A series of works were performed by Hou’s group to check the performance of these two methods [34,81]. According to their tests, the MM-GBSA method was applied to calculate inhibitor-BRD9 binding free energies with the following Equation (7):

(7) $$\Delta G_{bind}=\Delta E_{ele}+\Delta E_{vdw}+\Delta G_{gb}+\Delta G_{surf}-T\Delta S$$

in which $\Delta E_{ele}$ and $\Delta E_{vdw}$ individually indicate the electrostatic and van der Waals interactions of inhibitors with BRD9, while $\Delta G_{pol}$ and $\Delta G_{surf}$ separately suggest the polar and nonpolar contributions to solvent free energy of the inhibitor-BRD9 complexes. The $\Delta E_{ele}$ and $\Delta E_{vdw}$ were estimated from the Amber ff19SB force field parameters. The term $\Delta G_{surf}$ was estimated by using the empirical equation $\Delta G_{surf}=\gamma \times \Delta SASA+\beta$, in which ΔSASA denotes the solvent accessible surface area, and the parameters of γ and β are assigned as 0.0072 kcal·mol·Å^−2^ and 0.0 kcal·mol^−1^, respectively. The $\Delta G_{pol}$ was computed by using the generalized Born (GB) model [82]. The last component, −*T*∆*S*, represents the contribution of the entropic change to binding free energies and was estimated through the MMPBSA.py program in Amber 20 [83]. In our current work, 300 snapshots were extracted from the equilibration parts of MD trajectories to calculate binding free energies. Since the entropy calculation is computationally demanding, 100 snapshots were selected from the previously mentioned 300 to calculate the entropy contributions to inhibitor-BRD9 binding.

### 3.5. Solvated Interaction Energy Method

The SIE is another efficient method to quickly calculate binding free energies of inhibitors to targets. In this method, the SIE function [84] to estimate inhibitor-BRD9 binding free energies is expressed as the following Equation (8):

(8) $$\Delta G_{bind}(\rho ,D_{in},\alpha ,\gamma ,C)=\alpha \times [E_{c}(D_{in})+\Delta G^{R}+E_{vdW}+\gamma \cdot \Delta MSA(\rho )]+C$$

from which $E_{c}$ and $E_{vdW}$ correspond to the intermolecular Coulomb and van der Waals interaction energies in the bound state of BRD9, respectively. The component $\Delta G^{R}$ describes the change in the reaction field energy due to the binding of an inhibitor, which is obtained by solving the Poisson equation using the boundary element method (BRI BEM) [85,86] and a solvent probe with a variable radius of 1.4 Å [87]. The term γ·ΔMSA represents the change in the molecular surface area resulting from binding. The four empirical parameters used in this work, ρ, $D_{in}$, γ and C, represent the Amber van der Waals radii linear scaling coefficient, the solute interior dielectric constant, the molecular surface area coefficient and a constant, respectively. The empirical parameter α relates to the overall proportionality coefficient associated with the loss of conformational entropy upon binding [88]. The optimized values of these parameters are α=0.1048, $D_{in}=2.25$, ρ=1.1, γ=0.0129 kcal/(mol·Å) and $C=-2.89\;kcal\cdot {mol}^{-1}$ [84,89]. The SIE calculations were performed with the program Sietraj [89].

## 4. Conclusions

BRD9 is thought to be a key player in chromatin remodeling and gene expression regulation. Inhibition of BRD9 activity plays an important role in the treatment of certain cancers, making it a potential target for anticancer drugs. Three independent MD simulations were followed by Markov modeling and PCA to probe molecular mechanism of orthosteric and allosteric regulation on the activity of BRD9 by inhibitors 82I and POJ. Our results indicate that binding of orthosteric and allosteric inhibitors induces significant structural changes in the protein, particularly in the formation and dissolution of α-helical regions, and alters the transition pathway. Markov flux analysis reveals that notable changes occur in the α-helicity near the ZA loop during the inhibitor binding process. The calculations of binding free energies using the MM-GBSA method reveal that the cooperation of orthosteric and allosteric inhibitors affects the binding ability of inhibitors to BRD9 and alters the active sites of orthosteric and allosteric positions. This research is expected to provide new insights into the inhibitory mechanism of 82I and POJ on BRD9 and offer a theoretical foundation for the development of cancer treatment strategies targeting BRD9.

## Figures and Tables

**Table 1:** The flux analysis data of APO-BRD9.

Pathways	Path Flux (s^−1^)	Percentage of Total Coarse Flux (%)
SA → S1 → SB	2.09 × 10^−4^	98.9
SA → SB	1.22 × 10^−6^	0.6
SA → S1 → S2 → SB	1.20 × 10^−6^	0.6
Total	2.11 × 10^−4^	100

**Table 2:** Binding free energies of inhibitors to BRD9 obtained by MM-GBSA method.

Complex	82I-BRD9		POJ-BRD9		82I (ALL-BRD9)		POJ (ALL-BRD9)	
	Average	Std	Average	Std	Average	Std	Average	Std
$\Delta E_{ele}$	−10.96	6.42	−21.51	17.71	−9.27	7.60	−22.60	16.56
$\Delta E_{vdW}$	−23.37	5.20	−27.77	8.52	−18.50	8.54	−30.66	9.30
$\Delta G_{gb}$	17.59	5.46	30.09	16.44	15.06	7.63	32.76	14.66
$\Delta G_{surf}$	−3.02	0.62	−3.83	1.04	−2.45	1.09	−4.29	1.14
ΔGpola	6.63	5.94	8.58	17.08	5.79	7.62	10.16	15.61
−TΔS	14.94	3.84	20.60	4.68	13.85	5.24	21.19	3.88
ΔGbindb	−4.83		−2.42		−1.31		−3.59	

ΔaGpol=ΔEele+ΔGgb; ΔGbind=ΔEele+ΔGgb+ΔEvdw+ΔGsurf−TΔSb.

## Data Availability

Data is contained within the article and Supplementary Materials.

## Supplementary Materials

The following supporting information can be downloaded at: https://www.mdpi.com/article/10.3390/molecules29153496/s1. Table S1. The flux analysis data of 82I-BRD9; Table S2. The flux analysis data of POJ-BRD9; Table S3. The flux analysis data of ALL-BRD9; Table S4. Binding ability of inhibitors to BRD9 predicted by molecular docking; Table S5. Binding free energies of inhibitors to BRD9 obtained by MM-PBSA method; Table S6. Binding free energies of inhibitors to BRD9 obtained by SIE method; Figure S1. RMSDs, Rgs and MSA of the APO-, 82I-, POJ- and ALL-BRD9; Figure S2. RMSDs, Rgs and MSA of the 82I-, LIG-, P8Z- and POJ-BRD9; Figure S3. Secondary structure evolution of BRD9 in four systems over the simulation time; Figure S4. Structural flexibility of the 82I-, LIG-, P8Z- and POJ-BRD9; Figure S5. DCCMs of BRD9 calculated by using the coordinates of the Cα atoms; Figure S6. Concerted motions of structural domains in four systems; Figure S7. Percentage of macrostates for four systems accounting for the total sampling times after the equilibrium of systems; Figure S8. The distances between the ZA-loop and BC-loop; Figure S9. Binding poses of inhibitors to BRD9 predicted by molecular docking; Figure S10. Inhibitor-residue interactions in the inhibitor-bound BRD9.
