# Supplementary material for: Unveiling Allosteric Regulation and Binding Mechanism of BRD9 through Molecular Dynamics Simulations and Markov Modeling
Source: Molecules. 2024 Jul 25;29(15):3496. doi: 10.3390/molecules29153496 (PMC11314499; doi:10.3390/molecules29153496)
Supplement: Supplementary file 1 [file molecules-29-03496-s001.zip › molecules-3074384-supplementary.pdf]

Unveiling Allosteric Regulation and Binding Mechanism of BRD9 through Molecular Dynamics Simulations and Markov Modeling

Bin Wang [1], Jian Wang [2], Wanchun Yang [2], Lu Zhao [2], Benzheng Wei [1,*] and Jianzhong Chen [2,*]

[1] Center for Medical Artificial Intelligence, Shandong University of Traditional Chinese Medicine,
Qingdao 266112, China; wb1696843361@gmail.com

[2] School of Science, Shandong Jiaotong University, Jinan 250357, China;
wangjian_lxy@sdjtu.edu.cn (J.W.); yangwch1982@126.com (W.Y.);
zhaolusdu@163.com (L.Z.)

* Correspondence: wbz99@sina.com (B.W.); chenjianzhong1970@163.com (J.C.)

Table S1. The flux analysis data of 82I-BRD9

**Pathways**	**Path Flux(s^{-1})**	**Percentage of Total Coarse Flux(%)**
SA→S2→SB	2.81×10^{-4}	58.4
SA→S1→S2→SB	1.95×10^{-4}	40.5
SA→S3→SB	3.70×10^{-6}	0.8
SA→SB	8.30×10^{-7}	0.2
SA→S1→SB	5.49×10^{-7}	0.1
Total	4.82×10^{-4}	100

Table S2. The flux analysis data of POJ-BRD9

**Pathways**	**Path Flux(s^{-1})**	**Percentage of Total Coarse Flux(%)**
SA→S2→S1→SB	2.00×10^{-4}	51.1
SA→S2→S4→S1→SB	1.20×10^{-4}	30.6
SA→S1→SB	5.08×10^{-5}	13.0
SA→S4→SB	1.62×10^{-5}	4.1
SA→S3→S4→S1→SB	2.75×10^{-6}	0.7
SA→S4→S1→SB	1.16×10^{-6}	0.3
SA→S2→SB	6.64×10^{-7}	0.2
Total	3.91×10^{-4}	100

Table S3. The flux analysis data of ALL-BRD9.

Pathways	Path Flux(s^{-1})	Percentage of Total Coarse Flux(%)
SA→S1→SB	2.86×10^{-8}	76.2
SA→S1→S2→SB	7.35×10^{-9}	19.6
SA→S2→SB	1.58×10^{-9}	4.2
Total	3.75×10^{-8}	100

Table S4. Binding ability of inhibitors to BRD9 predicted by molecular docking.

Mode	Affinity(kcal/mol)			
	82I-BRD9	LIG-BRD9	P8Z-BRD9	POJ-BRD9
1	-5.2	-6.7	-7.1	-5.1
2	-4.4	-6.1	-7.0	-5.0
3	-4.1	-5.0	-5.7	-4.9
4	-3.9	-4.9	-5.7	-4.9
5	-3.9	-4.9	-5.5	-4.7
6	-3.8	-4.9	-5.3	-4.6
7	-3.6	-4.8	-5.3	-4.6
8	-3.6	-4.8	-5.3	-4.4
9	-3.6	-4.8	-5.2	-4.4
10	-3.5	-4.8	-5.2	-4.3
11	-3.5	-4.8	-5.1	-4.3
12	-3.5	-4.8	-5.0	-4.3
13	-3.4	-4.7	-5.0	-4.3
14	-3.4	-4.7	-4.9	-4.3
15	-3.3	-4.7	-4.9	-4.2
16	-3.3	-4.7	-4.9	-4.1
17	-3.2	-4.7	-4.9	-4.1
18	-3.2	-4.7	-4.8	-4.0
19	-3.2	-4.5	-4.8	-4.0
20	-3.0	-4.5	-4.8	-4.0

Table S5. Binding free energies of inhibitors to BRD9 obtained by MM-GBSA method.

Complex	82I-BRD9		LIG-BRD9		P8Z-BRD9		POJ-BRD9	
	**Average**	**Std**	**Average**	**Std**	**Average**	**Std**	**Average**	**Std**
ΔE_{ele}	-10.96	6.42	-8.37	4.75	-16.56	6.67	-21.51	17.71
ΔE_{vdW}	-23.37	5.20	-31.52	4.22	-35.82	5.74	-27.77	8.52
ΔG_{gb}	17.59	5.46	17.25	4.06	28.10	6.09	30.09	16.44
ΔG_{surf}	-3.02	0.62	-3.96	0.43	-4.58	0.70	-3.83	1.04
$^{a}\Delta G_{pol}$	6.63	5.94	8.88	4.40	11.54	6.36	8.58	17.08
$-T\Delta S$	14.94	3.84	17.39	3.56	13.85	5.24	20.60	4.68
$^{b}\Delta G_{bind}$	-4.83		-9.21		-9.71		-2.42	
$^{c}\Delta G_{exp}$	-6.28		-7.78		-9.14			

$^{a}\Delta G_{pol} = \Delta E_{ele} + \Delta G_{gb}$

$^{b}\Delta G_{bind} = \Delta E_{ele} + \Delta G_{gb} + \Delta E_{vdw} + \Delta G_{surf} - T\Delta S$

$^{c}\Delta G_{exp}$ The experimental values were derived from the experimental IC50 values in reference using the equation $\Delta G_{exp} = -RT\ln IC_{50}$

Table S6. Binding free energies of inhibitors to BRD9 obtained by SIE method.

**Complex**	**82I-BRD9**		**LIG-BRD9**		**P8Z-BRD9**		**POJ-BRD9**	
	**Average**	**Std**	**Average**	**Std**	**Average**	**Std**	**Average**	**Std**
ΔE_{vdm}	-23.37	5.20	-31.52	4.22	-35.82	5.74	-25.77	8.52
ΔE_{c}	-4.87	2.86	-3.72	2.11	-7.36	2.97	-7.57	7.88
ΔG^{R}	6.64	2.29	6.34	1.73	9.35	2.09	12.31	6.93
$\gamma \cdot \Delta MSA$	-4.32	0.97	-5.66	0.67	-6.68	1.00	-4.74	1.43
□ΔG_{bind}	-5.61		-6.51		-7.13		-5.59	
$^{a}\Delta G_{exp}$	-6.28		-7.78		-9.14			

$^{a}\Delta G_{exp}$ The experimental values were derived from the experimental IC50 values in reference using the equation $\Delta G_{exp} = -RT\ln IC_{50}$

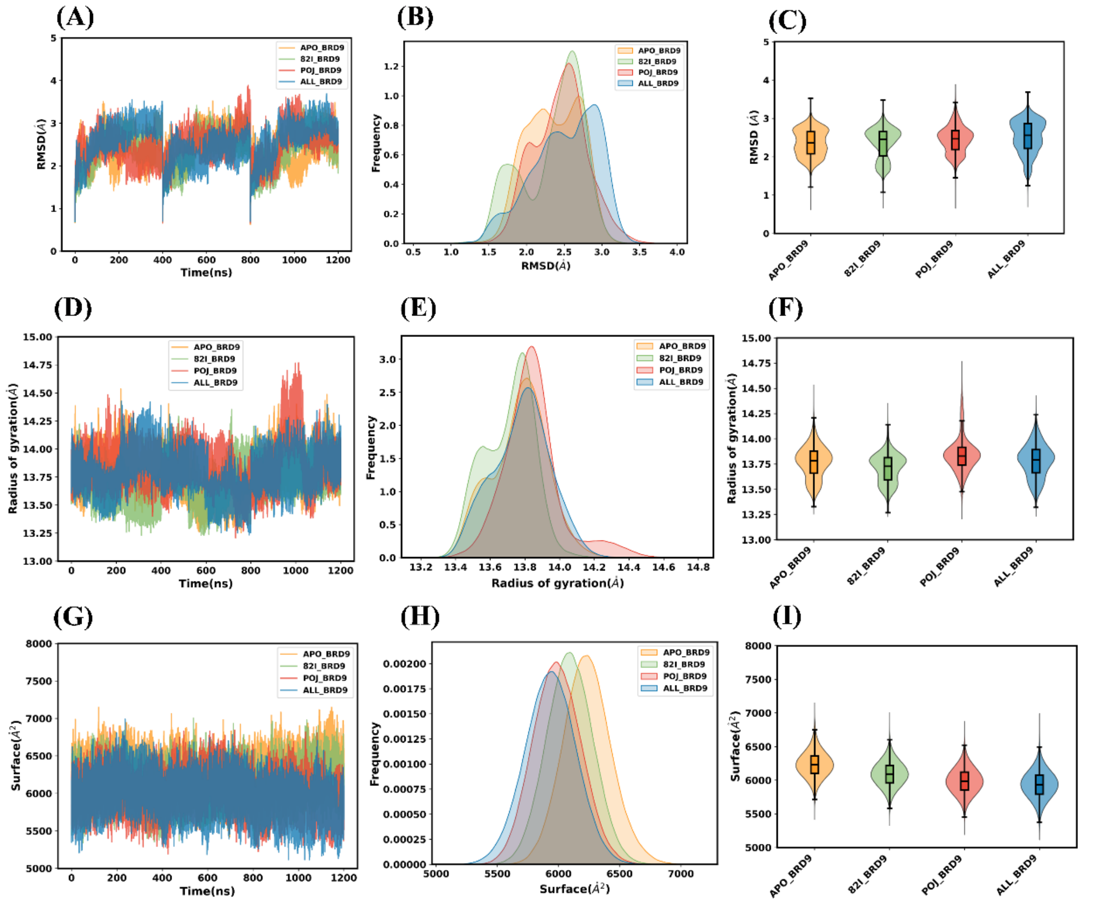

Figure S1. RMSDs, Rgs and MSA of the APO-, 82I-, POJ-, ALL-BRD9: (**A**) the time course of RMSDs, (**B**) the frequency distribution of RMSDs, (**C**) the violin distribution of RMSDs, (**D**) the time course of Rgs, (**E**) the frequency distribution of Rgs, (**F**) the violin distribution of Rgs, (**G**) the time course of MSAs, (**H**) the frequency of MSAs and (**I**) the violin distribution of MSAs.

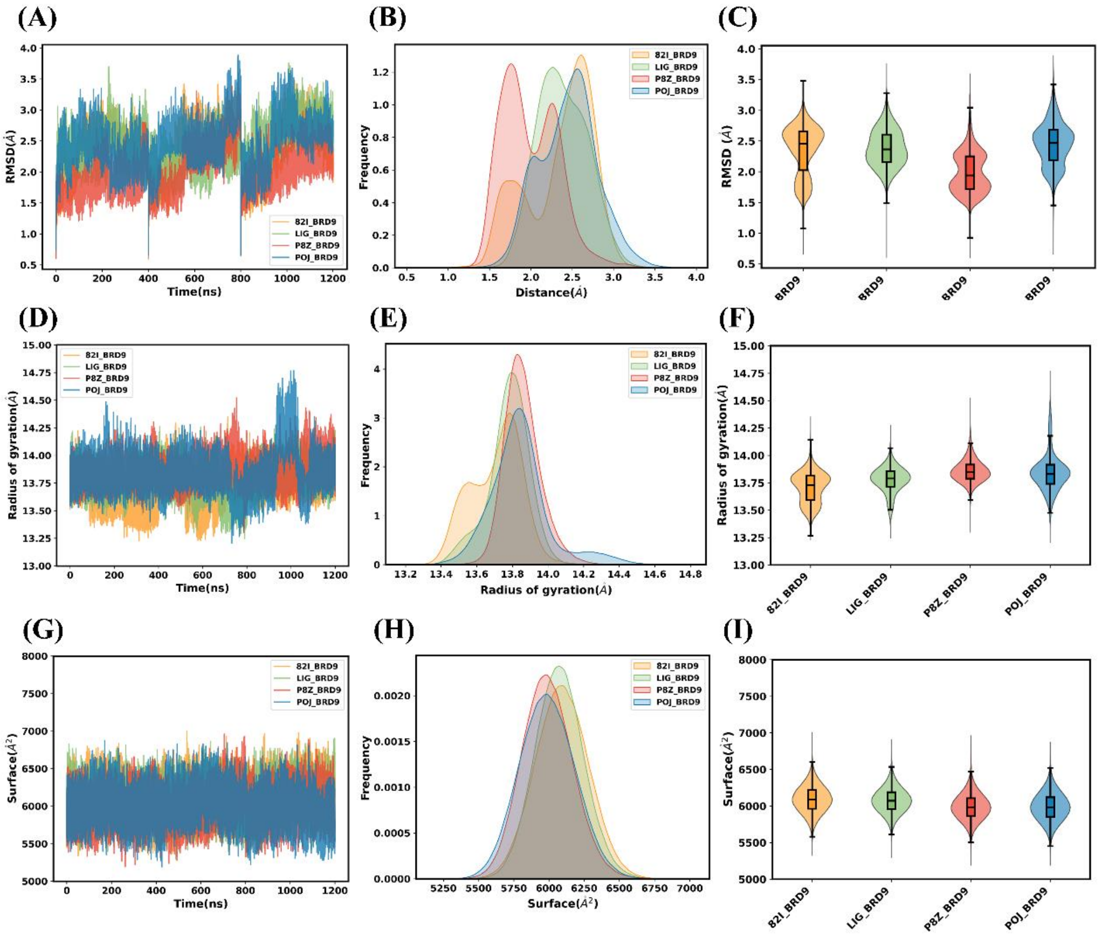

Figure S2. RMSDs, Rgs and MSA of the 82I-, LIG-, P8Z- and POJ-BRD9: (**A**) the time course of RMSDs , (**B**) the frequency distribution of RMSDs, (**C**) the violin distribution of RMSDs, (**D**) the time course of Rgs, (**E**) the frequency distribution of Rgs, (**F**) the violin distribution of Rgs, (**G**) the time course of MSAs, (**H**) the frequency of MSAs and (**I**) the violin distribution of MSAs.

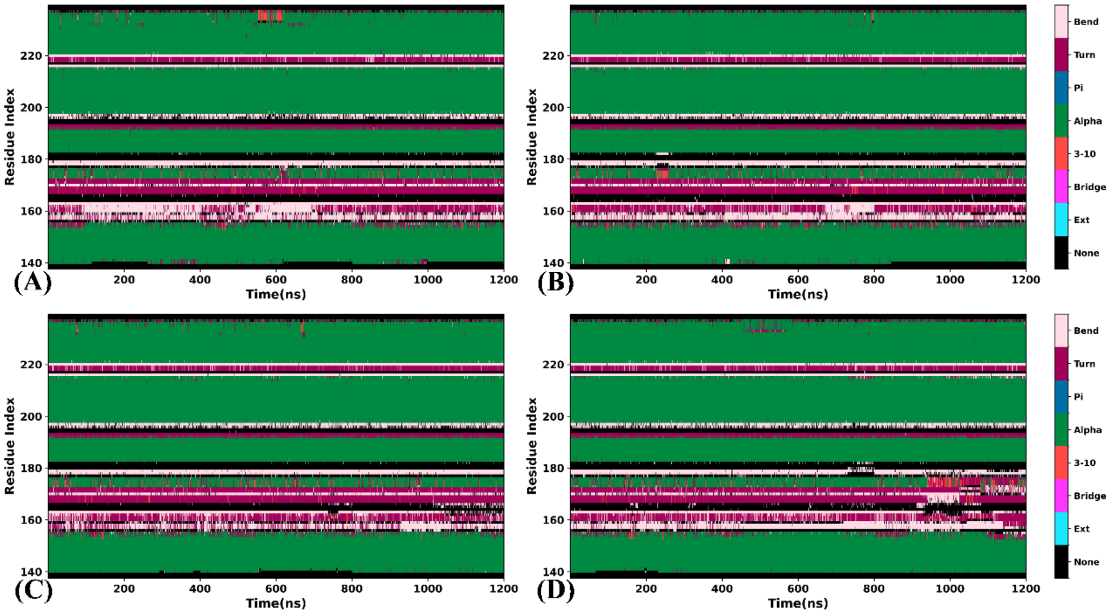

Figure S3. Secondary structure evolution of BRD9 in four systems over the simulation time: (**A**) the 82I-BRD9, (**B**) the LIG-BRD9, (**C**) the P8Z-BRD9 and (**D**) the POJ-BRD9.

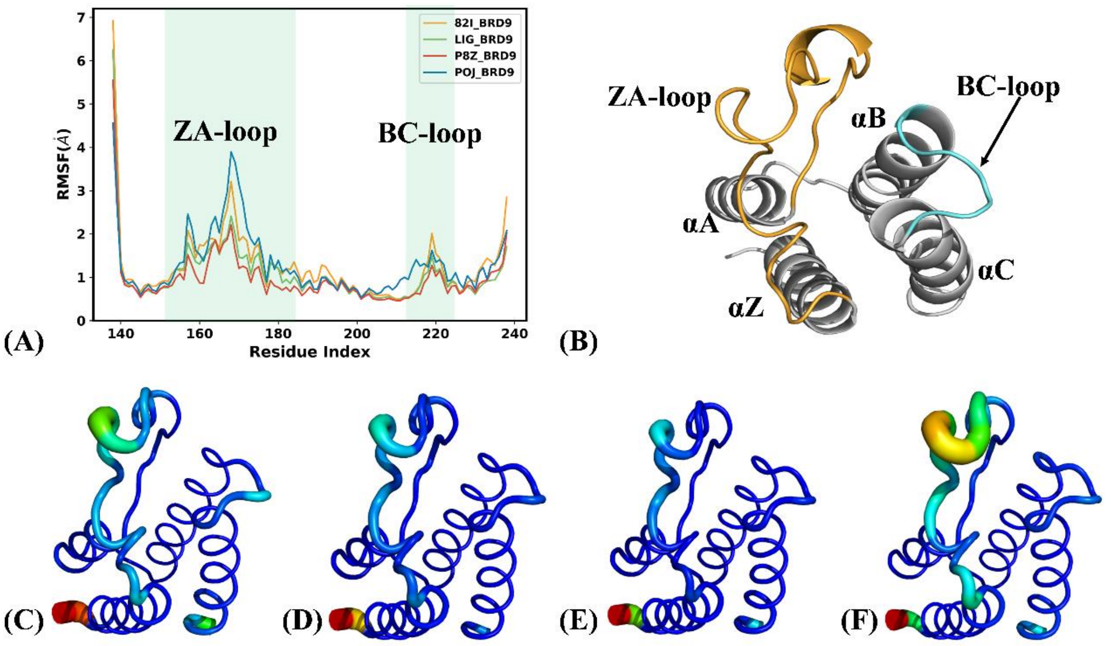

Figure S4. Structural flexibility of the 82I-, LIG-, P8Z-, POJ-BRD9: (**A**) the RMSFs of BRD9 calculated using the coordinates of the Cα atoms, (**B**) structural regions with obvious alterations of RMSFs and (**C–F**) corresponding to the structural flexibility of 82I-BRD9, LIG-BRD9, P8Z-BRD9 and POJ-BRD9. The tendency from blue to red indicates the increase of structural flexibility which is scaled in B-factor.

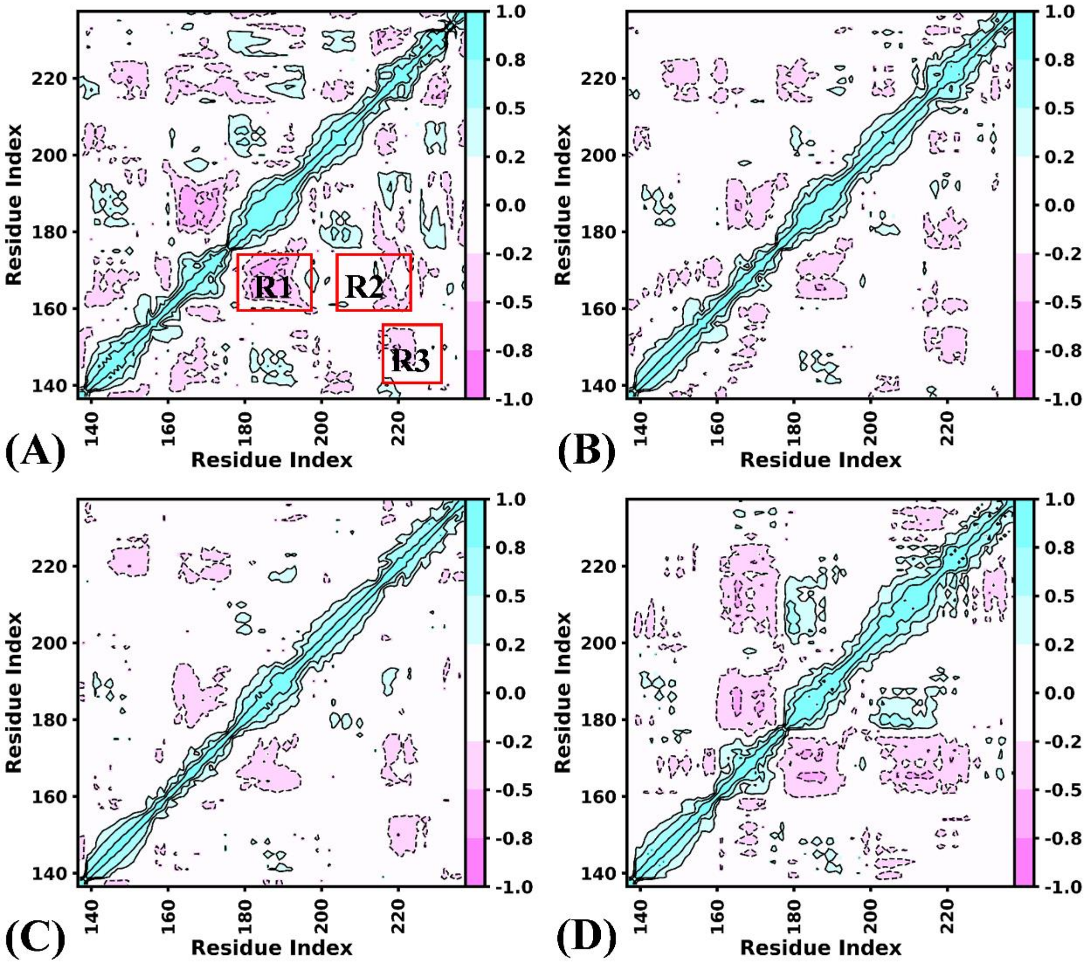

Figure S5. DCCMs of BRD9 calculated by using the coordinates of the Cα atoms: (**A**) the 82I-BRD9, (**B**) the LIG-BRD9, (**C**) the P8Z-BRD9 and (**D**) the POJ-BRD9.

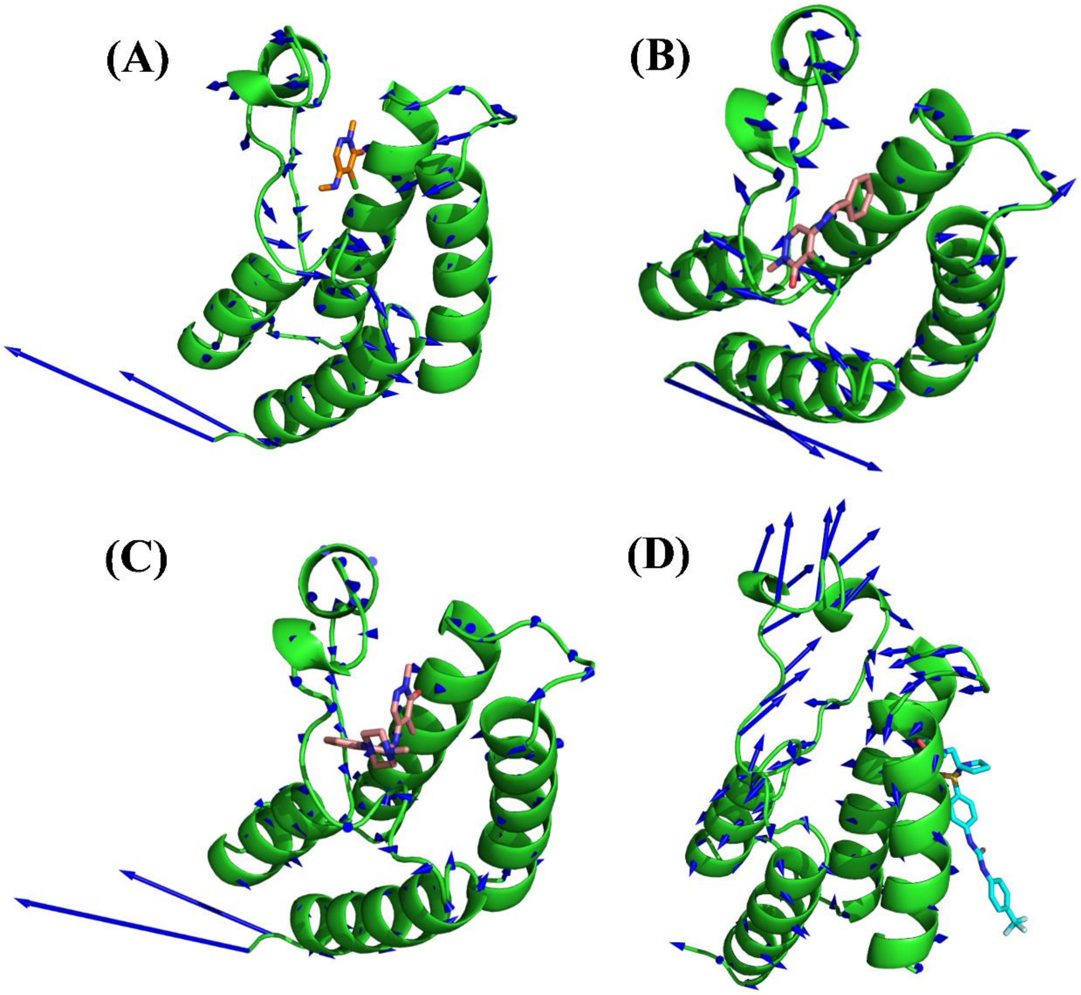

Figure S6. Concerted motions of structural domains in four systems: (**A**) the 82I-BRD9, (**B**) the LIG-BRD9, (**C**) the P8Z-BRD9 and (**D**) the POJ-BRD9. In this figure, BRD9 is shown in cartoon modes and inhibitors are displayed in stick modes.

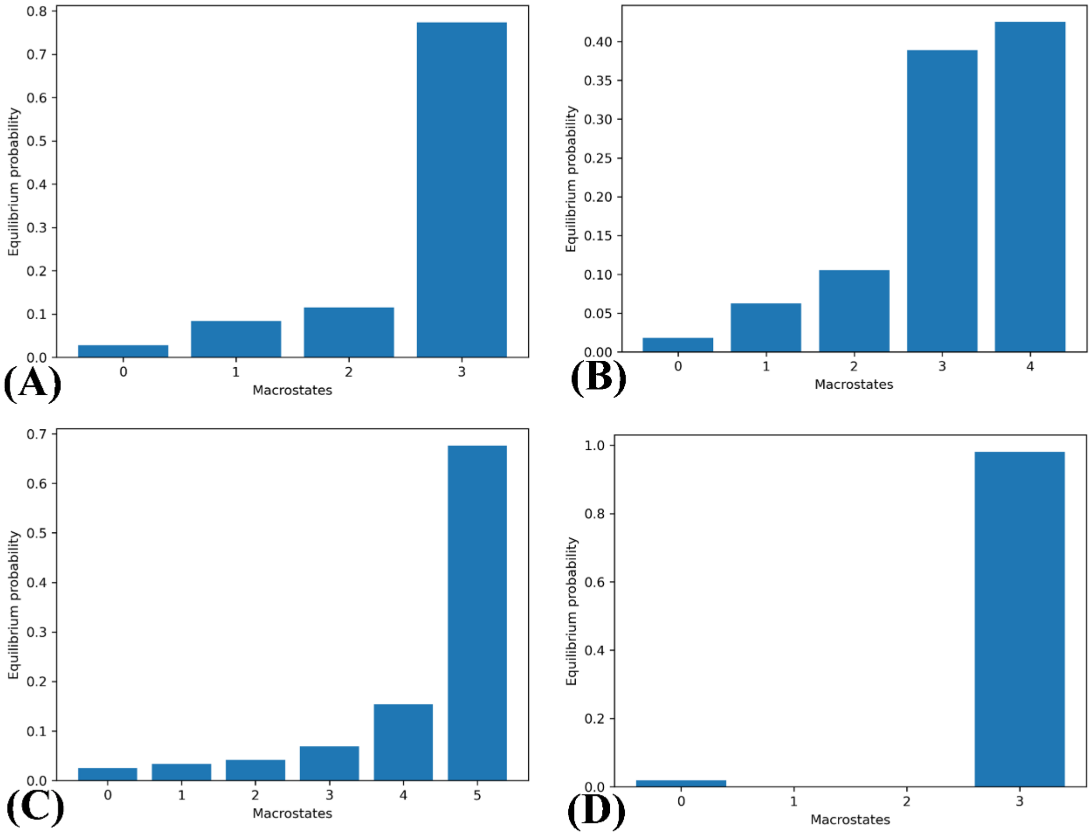

Figure S7. Percentage of macrostates for four systems accounting for the total sampling times after the equilibrium of systems: (**A**) the APO-BRD9, (**B**) the 82I-BRD9, (**C**) the POJ-BRD9 and (**D**) the ALL-BRD9.

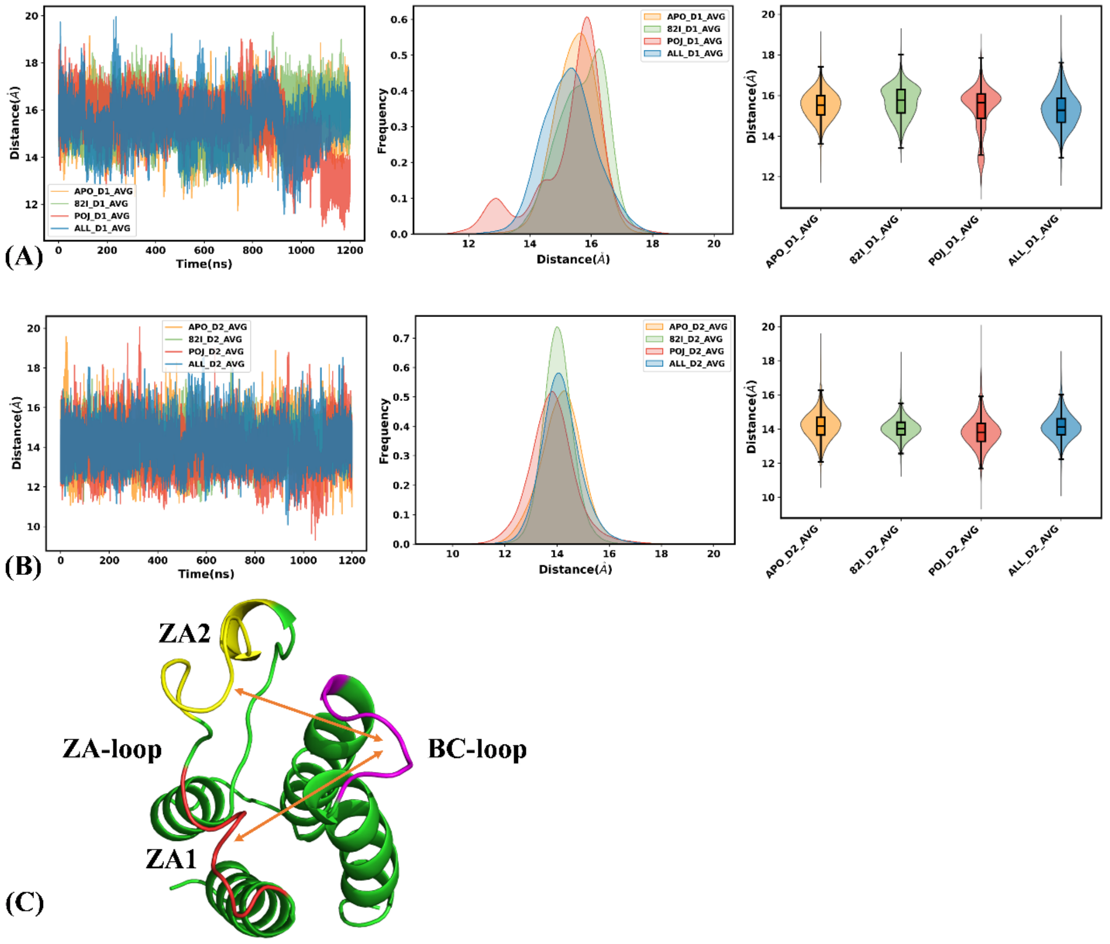

Figure S8. The distances between the ZA-loop and BC-loop: (**A**) the distance between the BC-loop and the ZA-loop segment ZA1 for the APO-BRD9, 82I-BRD9, POJ-BRD9 and ALL-BRD9. (**B**) The distance between the BC-loop and the ZA-loop segment ZA2 for the APO-BRD9, 82I-BRD9, POJ-BRD9, and ALL-BRD9 and (**C**) Schematic diagrams of the ZA1 and ZA2 domains of the ZA loop and their respective distances to the BC-loop.

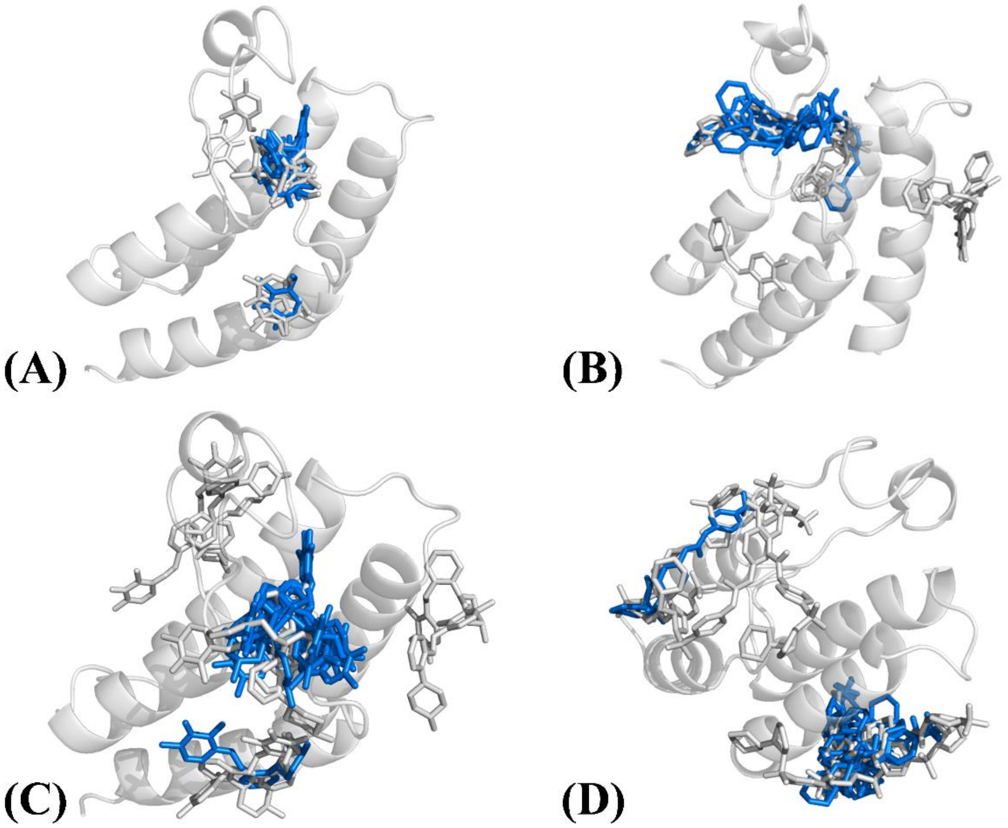

Figure S9. Binding poses of inhibitors to BRD9 predicted by molecular docking: (**A**) 82I, (**B**) LIG, (**C**) P8Z and (**D**) POJ. In this figure, inhibitors are shown in stick modes and BRD9 is displayed in cartoon styles. The structures with the first ten scoring are exhibited in the blue.

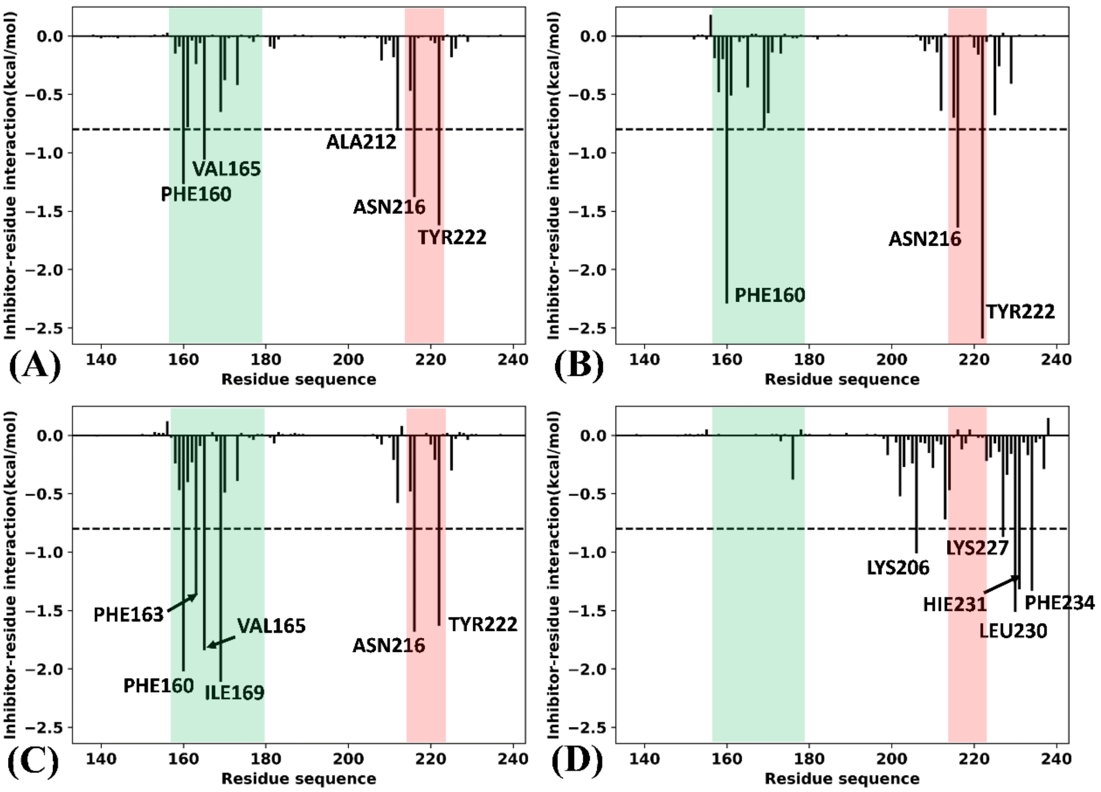

Figure S10. Inhibitor-residue interactions in the inhibitor-bound BRD9: (**A**) 82I, (**B**) LIG, (**C**) P8Z and (**D**) POJ.
